# Complete Genome Sequence of the Plant Growth-Promoting Rhizobacterium Pseudomonas fluorescens LBUM677

**DOI:** 10.1128/MRA.00438-19

**Published:** 2019-06-20

**Authors:** Amy Novinscak, David L. Joly, Martin Filion

**Affiliations:** a Biology Department, Université de Moncton, Moncton, New Brunswick, Canada; University of Southern California

## Abstract

Pseudomonas fluorescens LBUM677 has shown the ability to increase plant biomass and seed oil yield in soybean, canola, and Buglossoides arvensis (corn gromwell) when inoculated in the rhizosphere. Here, we report a draft genome sequence of P. fluorescens LBUM677, with an estimated size of 6.14 Mb.

## ANNOUNCEMENT

Bacteria from the Pseudomonas fluorescens species complex have long been known for their potential to promote plant growth (1). Pseudomonas fluorescens LBUM677 was isolated from the rhizosphere of strawberry plants by serial dilution of rhizosphere and subsequent plating on King agar B; it grows best in the laboratory at 25°C (2). This bacterium has shown the ability to increase plant biomass as well as oil and stearidonic acid (SDA) yields in Buglossoides arvensis (corn gromwell) (2). The oil from this plant is a viable alternative to the omega-3 fatty acids derived from marine sources (3). Additionally, a patent has been granted for this bacterium and its use for enhancing total lipid yields of soybeans (Glycine max) and canola (Brassica napus) (M. Filion and M. Surette, U.S. patent application 10,165,743). The ability of LBUM677 to increase oil yields in plants has yet to be pinpointed to specific genetic determinants; therefore, its genome was analyzed to help identify potential metabolic pathways involved in this process.

An individual colony of LBUM677 was used to inoculate Trypticase soy broth, which was then incubated for 48 h at 120 rpm and 25°C. Genomic DNA was extracted from the liquid culture using the UltraClean microbial DNA isolation kit (Qiagen, Germantown, MD) and purified using Agencourt AMPure XP beads (Beckman Coulter, Mississauga, Canada). Library preparation, DNA sequencing, and contig assembly were performed at the Génome Québec Innovation Centre (Montreal, Canada). Libraries were prepared with the 20-kb template preparation kit using the BluePippin size selection system protocol (Pacific Biosystems, Menlo Park, CA, USA). The DNA damage repair, end repair, and SMRTbell ligation steps were performed as described in the template preparation protocol with the SMRTbell template prep kit 1.0 reagents. DNA sequencing was done with the PacBio RS II single-molecule real-time (SMRT) platform using v1 chemistry. Three SMRT cells were used for sequencing, and the runs from all the cells were coassembled. Cell 1 generated 30,118 reads with an *N*_50_ value of 6,265 bp, cell 2 generated 63,198 reads with an *N*_50_ value of 7,098 bp, and cell 3 generated 101,969 reads with an *N*_50_ value of 7,665 bp. Assembly was performed using Hierarchical Genome Assembly Process (HGAP), generating a chromosome of 6,140,320 bp with an overall G+C content of 60.31% (119× coverage) (4). In the preassembly step, quality control was performed by aligning short reads on longer reads with BLASR (5); assembly was performed with Celera Assembler v8.1 (6), and polishing was performed with Quiver (4). Default settings were used for all software. The finalized genome was circularized manually using CLC Genomics Workbench v8.0 (CLC Bio, Boston, MA), and putatively ambiguous areas were visually inspected. Genome annotation was performed using the NCBI Prokaryotic Genome Annotation Pipeline, with default settings (7), and identified 5,406 predicted protein-coding sequences, 19 rRNA operons, and 71 tRNA loci.

**FIG 1:**
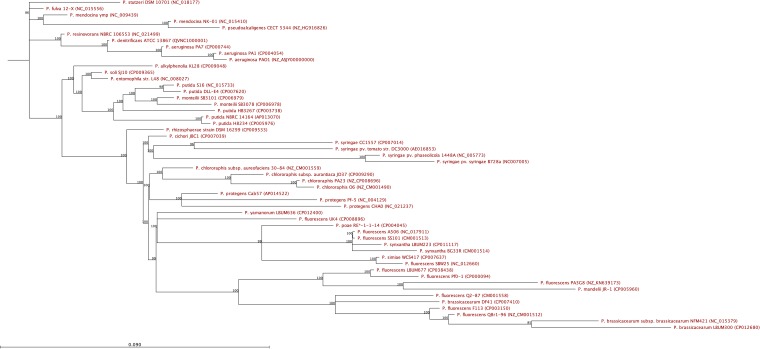
Neighbor-joining phylogenetic tree of 48 closely related *Pseudomonas* spp. with bootstrap values greater than 80% indicated at the nodes. The accession number of each strain is in parentheses.

Ten core housekeeping genes (*acsA*, *aroE*, *dnaE*, *guaA*, *gyrB*, *mutL*, *ppsA*, *pyrC*, *recA*, and *rpoB*) from 48 closely related *Pseudomonas* spp. were retrieved from DDBJ/ENA/GenBank and compared based on concatenated alignments using the CLC Genomics Workbench v8.0. The resulting neighbor-joining phylogenetic tree (Fig. 1) indicated that P. fluorescens LBUM677’s closest relative is P. fluorescens Pf0-1 and that it belongs to the P. fluorescens subclade 2, according to the classification by Loper et al. (8).

### Data availability.

This whole-genome shotgun project has been deposited in DDBJ/EMBL/GenBank under the accession number CP038438. The version described in this paper is the first version. The accession number of the original read data set in the SRA is SRX5620962.
